# TLR5 agonist in combination with anti-PD-1 treatment enhances anti-tumor effect through M1/M2 macrophage polarization shift and CD8^+^ T cell priming

**DOI:** 10.1007/s00262-024-03679-5

**Published:** 2024-04-17

**Authors:** Junseok Lee, Keon-Il Im, Sojin Gil, Hyemin Na, Gi-June Min, Nayoun Kim, Seok-Goo Cho

**Affiliations:** 1 Institute for Translational Research and Molecular Imaging, The Catholic University of Korea, Seoul, Republic of Korea; 2 Department of Biomedicine and Health Sciences, College of Medicine, The Catholic University of Korea, Seoul, Republic of Korea; 3 Department of Hematology, Seoul St. Mary’s Hematology Hospital, College of Medicine, The Catholic University of Korea, Seoul, Republic of Korea

**Keywords:** TLR5, Immune checkpoint, PD-1, Tumor-associated macrophages, Cancer immunotherapy

## Abstract

Immune checkpoint inhibitors have revolutionized anti-tumor therapy, notably improving treatment responses in various tumors. However, many patients remain non-responsive and do not experience benefits. Given that Toll-like receptors (TLRs) can counteract tumor immune tolerance by stimulating both innate and adaptive immune responses, TLR agonists are being explored as potential immune adjuvants for cancer treatment. In this study, we assessed the potential of enhancing the efficacy of immune checkpoint inhibitors by activating innate immunity with a TLR5 agonist. In a mouse tumor model, combination therapy with TLR5 agonist and anti-PD-1 significantly inhibited tumor growth. The TLR5 agonist shifted the balance from M2-like to M1-like macrophages and upregulated the expression of co-stimulatory molecules in macrophages. Furthermore, TLR5 agonist promoted the activation and tumor infiltration of CD8^+^ T cells. As a result, the TLR5 agonist augmented the anti-tumor efficacy of anti-PD-1, suggesting its potential in modulating the tumor microenvironment to enhance the anti-tumor response. Our findings point toward the possibility of optimizing immune checkpoint inhibitor therapy using TLR5 agonists.

**Supplementary Information:**

The online version contains supplementary material available at 10.1007/s00262-024-03679-5.

## Introduction

The recent development of immune checkpoint inhibitors, including monoclonal antibodies (mAbs) targeting the programmed cell death receptor 1 (PD-1) and cytotoxic T-lymphocyte antigen-4 (CTLA-4), has markedly improved treatment responses in tumor patients [1]. Nevertheless, a significant number of patients fail to benefit from these treatments due to issues like primary resistance, where the cancer does not respond initially, and acquired resistance, in which cancers that initially responded rapidly relapse and progress [2].

The tumor microenvironment (TME) is an extrinsic factor leading to resistance against immunotherapy, including immune checkpoint inhibitors. Within the TME, factors like the absence of T cells and the presence of immunosuppressive entities, such as tumor-associated macrophages (TAMs), regulatory T cells (Tregs), and MDSCs, disrupt the anti-tumor immune response [3]. The efficacy of immunotherapy has a positive correlation with CD8^+^ T cell expression [2]. Elevated levels of CD8^+^ T cells within the TME predict a more favorable prognosis for patients with various solid tumors [4, 5]. Another determinant, TAMs, interface with CD8^+^ T cells, obstructing their migration and infiltration into tumors [6]. TAMs can be categorized as M1- or M2 macrophages, distinguished based on surface molecule expression and cytokine profiles. M2 macrophages release vascular epithelial growth factor (VEGF) and immunosuppressive molecules, like IL10 and TGFβ, facilitating tumor growth [7]. Eliminating M2 macrophages has been shown to decrease tumor growth in multiple mouse tumor models, such as breast cancer and melanoma [8, 9]. Conversely, M1-like macrophages discharge pro-inflammatory cytokines like TNF-*α* and IL-1*β*, prompting inflammatory reactions and attacking cancer cells [7]. Patients displaying a higher M1/M2 TAM ratio experienced a better 5-year prognosis [10]. Given the adaptability of macrophages, transforming M2–M1 macrophage is a viable strategy to amplify the anti-tumor immune response.

Toll-like receptors (TLRs), as notable pattern recognition receptors (PRRs), activate both innate and adaptive immune responses by detecting pathogen-associated molecular patterns (PAMPs), which are conserved molecular motifs from microbes [11, 12]. Expressed predominantly in epithelial cells and innate immune cells like macrophages and dendritic cells (DCs) [13, 14], TLRs combat tumor immune tolerance by enhancing innate immunity within the TME. Consequently, TLR agonists are being explored as potential immune adjuvants in cancer treatments [15, 16]. Specifically, TLR5 is mainly found in DCs in the lamina propria and intestinal epithelial cells, orchestrating the innate immune response upon recognizing bacterial flagellin [17–19]. High TLR5 expression has been documented in breast and gastric carcinoma cells [20, 21]. Recognizing the significance of TLR5 in initiating innate immunity, numerous studies have scrutinized TLR5's potential as an immune adjuvant [22]. Furthermore, TLR5 agonists demonstrated robust anti-tumor actions in tumor models, including breast, colon cancer, and melanoma [21, 23, 24].

KMRC011 is a novel TLR5 agonist derived from Salmonella flagellin-based radiation countermeasures by CONNEXT Co. Ltd. (Yeongcheon, Korea). Traditionally, the landscape of TLR5 agonists has comprised natural flagellin and recombinant protein derivatives such as entolimod (CBLB502), formulated by Cleveland BioLabs in the United States. This formulation is based on the conserved flagellin domain, which interacts directly with the innate immune receptor TLR5 [25]. KMRC011 retains the critical D0 and D1 domains of flagellin, which are essential for TLR5 binding, thereby ensuring its efficacy as an agonist. Addressing the potential for toxic immunogenic reactions associated with the artificially introduced N-terminal region in entolimod, KMRC011 was designed without this ancillary region [26]. Therefore, KMRC011 may have improved safety and a reduced risk of adverse effects compared with entolimod, although no comparative clinical studies have been conducted.

We hypothesized that TLR5 agonists’ activation of innate immune cells might prime CD8^+^ T cells and promote their migration and tumor infiltration, subsequently enhancing the efficiency of immune checkpoint inhibitors. Our findings indicate that combining TLR5 treatment with an anti-PD-1 antibody (Ab) synergistically curtails tumor growth. TLR5 agonists promote a shift from M2-like macrophages to M1-like macrophages and activate CD8^+^ T cells, suggesting their potential to enhance the effectiveness of PD-1 inhibitors in mouse tumor models.

## Materials and methods

### Cell culture and reagents

The mouse colon carcinoma cell lines MC-38 was obtained from Korean Cell Line Bank (KCLB) and CT-26 was obtained from ATCC. Cells were routinely cultured in RPMI and DMEM medium containing 10% fetal calf serum. Cells were maintained at 37 °C in a humidified atmosphere of 5% CO2. KMRC011, a TLR5 agonist, was developed and supplied by Connext ([Daegu], Korea). KMRC011 is a biologically recombinant protein derived from Salmonella enterica flagellin. While it retains the TLR5 binding domain of Salmonella enterica flagellin, the N-terminal ancillary tail has been removed to prevent unnecessary immune responses. Anti-PD-1, anti-CD8 and anti-CD4 were sourced from Bio X Cell (Lebanon, NH, USA).

### Animals

Female C57BL/6 mice aged between 5 and 8 weeks were procured from OrientBio (Sungnam, Korea). These mice were housed under specific pathogen-free conditions in an animal facility, with a humidity of 55% ± 5%, a 12/12 h light/dark cycle, and a temperature of 22 ± 1 °C. The facility’s air underwent filtration through a HEPA system. Mice had ad libitum access to mouse chow and tap water. All animal research procedures adhered to the Laboratory Animals Welfare Act, the Guide for the Care and Use of Laboratory Animals, and the Guidelines and Policies for Rodent Experiments provided by the IACUC (Institutional Animal Care and Use Committee) of the School of Medicine at The Catholic University of Korea (Approval number: CUMS-2018-0258-01). The IACUC received full accreditation from AAALAC International in 2018.

### MC-38 tumor induction

Mice received a subcutaneous (s.c.) injection with MC-38 cells (1 × 10^6^/200 μL) into their shaved right flank. Once tumors reached a size of 0.1–0.2 cm (typically palpable by day 6), mice were given an intraperitoneal (i.p.) injection of either 200 μL of TLR agonist (100 μg/kg) or anti-PD-1 (200 μg/mouse). Treatments were delivered once every 3 days, for a total of three sessions. Tumors were measured daily using calipers and data are expressed as means ± SEM.

### Flow cytometric analysis

Tumors were dissociated using a mouse tumor dissociation kit with the gentleMACS Octo Dissociator according to the manufacture’s protocol (Miltenyi Biotec). Draining lymph node (dLN) cells and spleen cells were dissociated in RPMI1640 supplemented with 5% FBS. Tumor infiltrating immune cells, dLN cells and spleen cells were prepared as single cell suspensions and immunostained with various combinations of the following fluorescence-conjugated Abs: CD3, CD4, CD8, DX5, F4/80, CD11c, CD206, CD40, CD80, PD-L1, IFN- γ, and granzyme B. Intracellular markers were identified using Abs for IFN-γ and granzyme B (eBioscience, San Diego, CA, USA). Prior to intracellular cytokine staining, cells were incubated in culture medium containing PMA (25 ng/ml; Sigma-Aldrich, St. Louis, MO, USA), ionomycin (250 ng/ml; Sigma-Aldrich), and monensin (GolgiStop, 1 μl/ml; BD Biosciences, Franklin Lakes, NJ, USA) in a 5% CO_2_ atmosphere at 37 °C for 4 h. Intracellular staining utilized an intracellular staining kit (eBioscience) as per the manufacturer’s guidelines. Analysis was conducted using a FACS LSR Fortessa cytometer (BD Biosciences) and FlowJo software (BD Biosciences).

### Immunohistochemistry

Tissues were fixed in formalin, embedded in paraffin, and then sectioned to a thickness of 3 μm. For immunohistochemistry, slides underwent dehydration with xylene and ethanol, followed by antigen retrieval and blocking. Sections were subsequently incubated with primary Abs Ki67 (1:200; Abcam, Cambridge, UK) or cleaved caspase-3 (1:100; Abcam) overnight at 4 °C. The secondary Ab used was anti-rabbit IgG-HRP (Santa Cruz Biotechnology, Dallas, TX, USA), with slides incubated at room temperature for 2 h. Detection of signals was performed using the REAL EnVision detection system, peroxidase/DAB (Dako, Santa Clara, CA, USA). Sections were counterstained using Mayer’s hematoxylin (Dako) for 1 min at room temperature. The quantification analyses of immunohistochemical staining were performed using ImageJ Software.

### Cytotoxicity assays

Furthermore, we performed an in vitro cytotoxicity assay using the CT-26 cell line to verify the results of our study with a different cell line. BALB/c splenocytes were plated with 1 μg/mL anti-CD28 antibody in 48-well plates previously coated with 1 µg/mL anti-CD3 antibody for 48 h at 37 °C, in the presence of TLR5 agonist (100 ng/ml) and/or anti-PD-1 abs (10ug/ml) (or vehicle treatments) prior to cytotoxicity assays. CT-26 tumor cells stained with CellTrace CFSE (5 μM) (Thermo Fisher) and treated with TLR5 agonist (100 ng/ml) and/or anti-PD-1 abs (10ug/ml) (or vehicle treatments) for 24 h. On the day of the assay, Pre-treated splenocytes were added to the CT-26 tumor cells at a 1:20 (target:effector) ratio. Cells were stained with Propidium Iodide (Thermo Fisher) and cytotoxicity was determined by flow cytometry.

### Statistical analysis

Data are presented as means ± standard error mean (SEM) unless otherwise noted. Comparisons between two groups were performed using the Mann–Whitney *U* test or Student’s *t* test, while for comparisons among multiple groups the Kruskal–Wallis test was used. Statistical analyses were conducted with SPSS Statistics software (version 16.0; IBM Corp., Armonk, NY, USA). *P* values < 0.05 were considered statistically significant.

## Results

### Combination therapy with TLR5 agonist and systemic anti–PD-1 Ab synergistically inhibits tumor growth

**Fig. 1:**
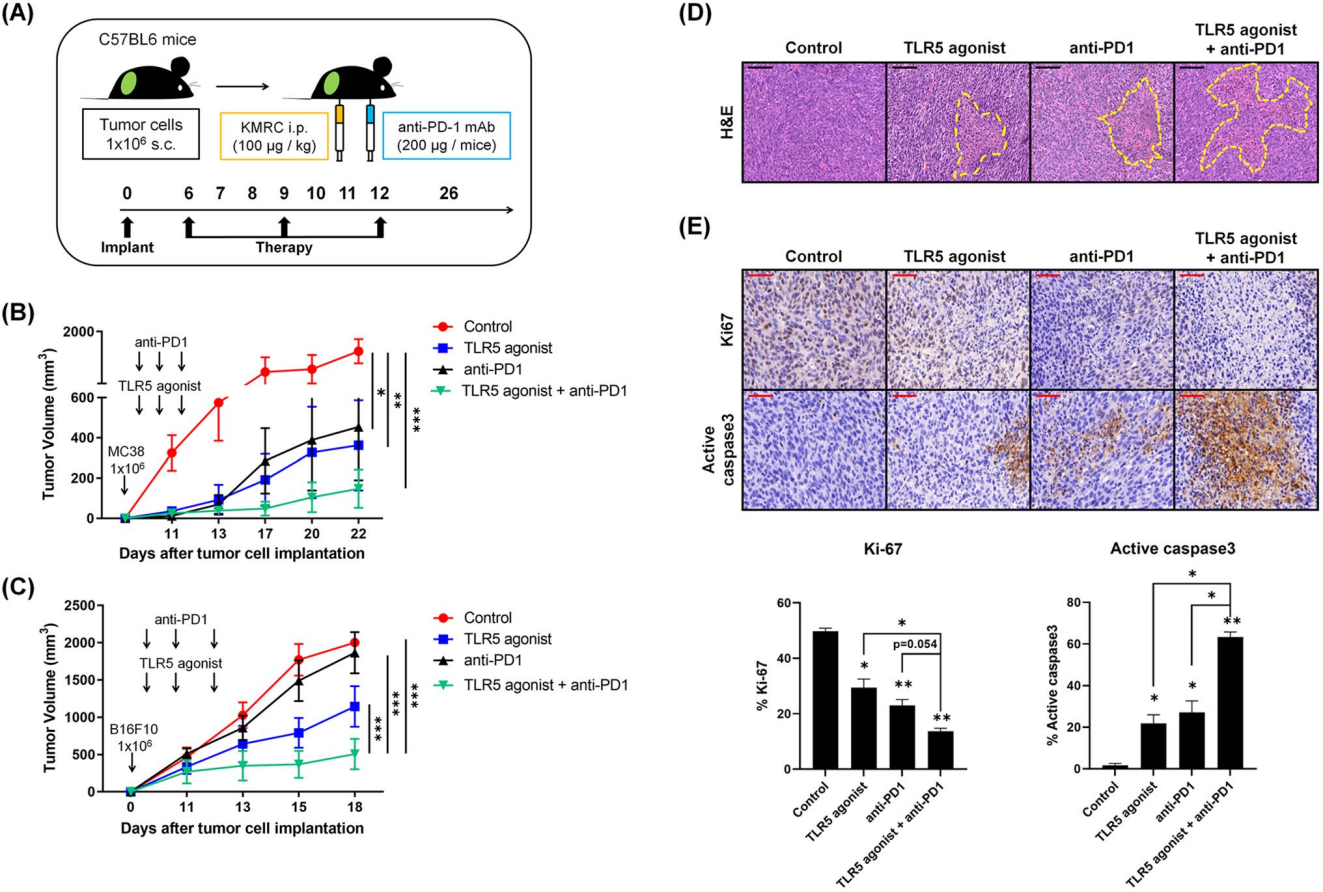
Combination therapy with TLR5 agonist and systemic anti–PD-1 antibody synergistically inhibits tumor growth. **A** MC-38 or B16F10 cells (1 × 106) were subcutaneously (s.c.) implanted in the flanks of C57BL/6 mice (*n* = 8/group). On day 6, mice received either TLR5 agonist (100 µg/kg), anti-PD-1 (200 µg/mice), or a combination of both, administered once every 3 days (three times in total). (B, C) Changes in tumor volume across groups in **B** MC-38 tumor model and **C** B16F10 tumor model. Tumor size was measured every 2–4 days starting on day 11. **D** Representative tumor histology. MC-38 tumors collected on day 26 after implantation were H&E stained. Scale bars, 100 µm. **E** Representative Immunohistochemical (IHC) staining of Ki67 and active caspase-3 in tumors. Scale bars, 50 µm. Tumors harvested on day 26 after implantation were stained for Ki67 and active caspase-3. Quantification of (mean ± SEM) of Ki67 and active caspase-3 is shown. *p < 0.05, **p < 0.01, ***p < 0.001. Results are representative of three independent experiments (*n* = 3/group)

We assessed the potential of TLR5 agonists and anti-PD-1 as monotherapies or combination therapy to suppress tumor growth. MC-38 cells were introduced into C57BL6 mice, which were then categorized into four groups based on their treatment: TLR5 agonist (100 µg/kg), anti-PD-1 (200 µg/mice), TLR5 agonist combined with anti-PD-1, and PBS (control). Treatment began 6 days after tumor implantation and was administered every 3 days for a total of three rounds (Fig. 1A). In a preliminary experiment, we compared the control group with the isotype control antibody (RatIgG2a, k); however, no difference was seen between the two groups and thus the isotype control arm was not included in the main study (Supplementary Fig. 1). Subsequently, we tracked changes in tumor volume (Fig. 1B). Both the TLR agonist and anti–PD-1 monotherapies suppressed tumor growth (Fig. 1B, Supplementary Fig. 2). Notably, the combination therapy further enhanced this suppressive effect. We conducted additional experiments using the B16F10 tumor model in C57BL6 mice to validate the efficacy of TLR5 agonist and anti-PD-1 combination therapy in suppressing tumor growth. Consistent with findings in the MC-38 model, we observed that TLR5 agonist monotherapy inhibited tumor growth, whereas anti-PD-1 monotherapy did not show significant effects (Fig. 1C, Supplementary Fig. 2). Importantly, combination therapy with the TLR5 agonist and anti-PD-1 further enhanced the suppressive effect on tumor growth. On day 23 after tumor implantation, tumors were extracted and examined for necrosis using H&E staining. The combination therapy group exhibited the most extensive necrosis in H&E-stained tumor sections (Fig. 1D). To evaluate tumor cell proliferation and apoptosis, we analyzed Ki67 (a proliferation marker) and caspase-3 (an apoptosis marker) expression in the tumor tissues through immunohistochemistry. The combination treatment significantly reduced Ki67 expression while elevating activated caspase-3 expression (Fig. 1E). This indicates that combining TLR5 agonists with anti–PD-1 magnifies the anti-tumor effectiveness of anti–PD-1.

### TLR5 agonist or combination treatment promote a shift from M2-like macrophages to M1-like macrophages

**Fig. 2:**
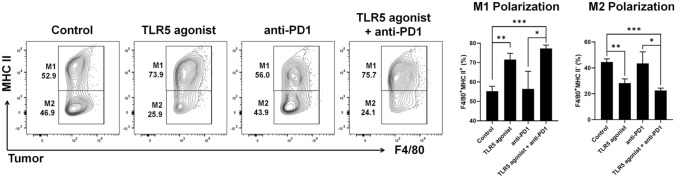
TLR5 agonist or combination treatment promotes a shift from M2-like macrophages to M1-like macrophages in the tumor. Flow cytometry analyses of M1-like (F4/80^+^MHC II^+^) and M2-like (F4/80^+^MHC II^−^) macrophages in tumors from tumor-bearing mice. Tumor tissue was sampled on day 11 after tumor implantation in the MC-38 tumor model. Bars represent means ± SEM. *p < 0.05, **p < 0.01, ***p < 0.001. The results are representative of three independent experiments (*n* = 3/group)

Previous studies have reported that bacterial flagellin, a TLR5 agonist, induces the polarization of M1-like macrophages [27]. Similarly, we hypothesized that KMRC011 might promote a shift from M2-like macrophages to M1-like macrophages. On day 11, tumor-infiltrating immune cells were harvested and the populations of M1-like (F4/80^+^MHC II^+^) and M2-like (F4/80^+^MHC II^−^) macrophages were quantified using flow cytometry. Both the TLR5 agonist monotherapy and combination therapy groups showed a marked reduction in the M2-like macrophage population, with a corresponding increase in M1-like macrophages. By contrast, the anti-PD-1 monotherapy group had M1-like and M2-like macrophage levels similar to the control (Fig. 2). The identified M1-like macrophages expressed TNF-a but did not express IL-10 (Data not shown). These results were similar in both the spleen and LN (Supplementary Fig. 3). These findings suggest that TLR5 agonist therapy induces a macrophage-polarization shift.

### TLR5 agonist or combination treatment increases co-stimulatory molecules on macrophages

**Fig. 3:**
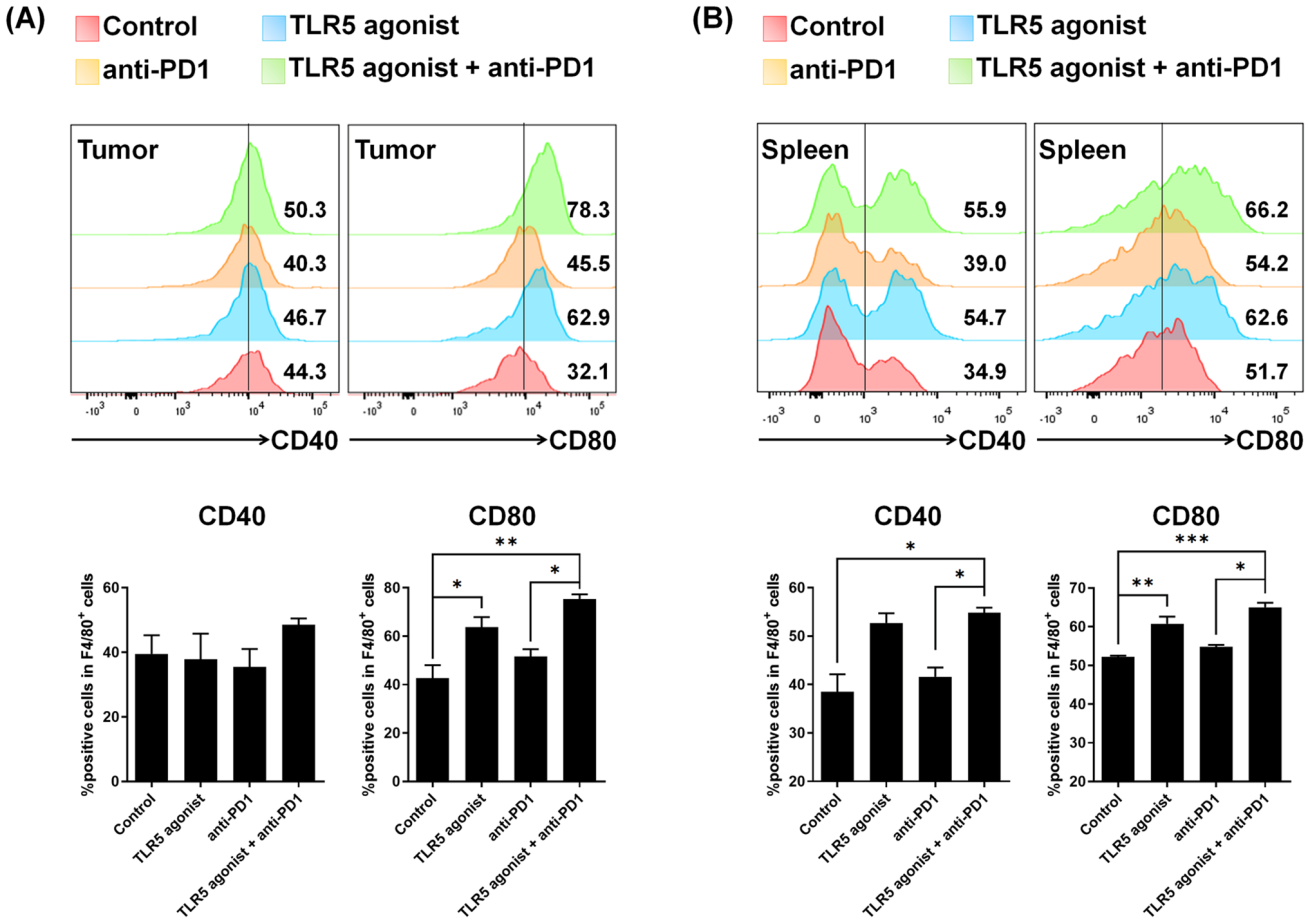
TLR5 agonist or combination treatment elevates co-stimulatory molecule expression in macrophages. (A, B) Flow cytometry analyses of co-stimulatory molecules (CD40 and CD80) on macrophages from the **A** tumor and **B** spleen tissues of tumor-bearing mice. Tumor tissues were sampled on day 11 after tumor implantation in the MC-38 tumor model. Spleen tissues were sampled on day 23 after tumor implantation in the MC-38 tumor model. Bars represent means ± SEM. *p < 0.05, **p < 0.01, ***p < 0.001. The results are representative of three independent experiments (*n* = 3/group)

To delve deeper into the effect of TLR5 agonist treatment on macrophage function, particularly the activation of T cell immune responses, we examined the co-stimulatory molecules CD40 and CD80 on macrophages from the tumor and spleen. We observed an increase in F4/80^+^CD80^+^ macrophages in the tumor and spleen from the TLR5 agonist monotherapy and combination therapy groups (Fig. 3A, B). Moreover, the population of F4/80^+^CD40^+^ macrophages increased in spleen of these groups; whereas, the difference in the tumor was not significant. These results were similar in LN (Supplementary Fig. 4).

### ***TLR5 agonist or combination treatment increases the activated CD8***^+^***population***

**Fig. 4:**
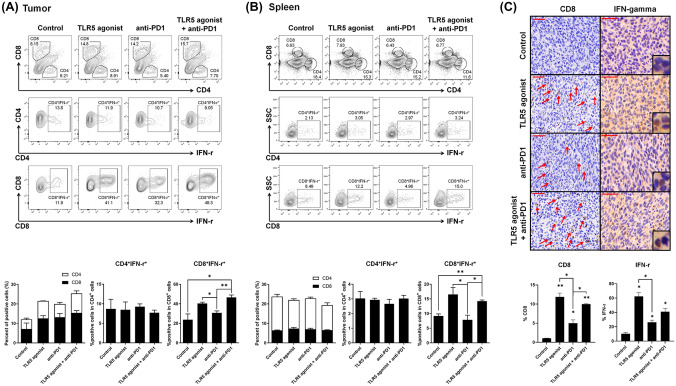
TLR5 agonist or combination treatment increases the activated CD8^+^ population in the tumor and spleen. (A, B) Flow cytometry analyses of T cells (CD4^+^ IFN-γ^+^, CD8^+^ IFN-γ^+^) in **A** tumors and **B** spleens of tumor-bearing mice. Tumor tissues were sampled on day 11 after tumor implantation in the MC-38 tumor model. Spleen tissues were sampled on day 23 after tumor implantation in the MC-38 tumor model. **C** Representative IHC staining of infiltrated CD8^+^ T cells and IFN-γ in tumors. Scale bars, 50 µm. The CD8^+^ T cells and IFN-γ are quantified (mean ± SD). Tumors harvested on day 23 after implantation were stained for CD8 and IFN-γ. Bars indicate means ± SEM. *p < 0.05, **p < 0.01. The results are representative of three independent experiments (*n* = 3/group)

To ascertain whether macrophage activation by TLR5 agonist treatment promotes the activation and tumor infiltration of CD8^+^ T and NK cells, tumor-infiltrating immune cells and splenocytes from tumor-bearing mice were analyzed using flow cytometry. While the total CD8^+^ population remained unchanged with TLR5 agonist monotherapy and combination therapy, the activated CD8^+^ T cell subset expressing cytokine IFN-γ increased significantly in both TLR agonist-treated groups (Fig. 4A, B), in both the tumor and spleen. Similarly, the activated NK cell subset expressing cytokine IFN-γ also increased in the combination therapy groups (Supplementary Fig. 5). Consistent with these data, IHC analysis of tumor tissues revealed that tumor infiltration by CD8^+^ T cells and cytokine IFN-γ expression were elevated with TLR5 agonist monotherapy and combination therapy (Fig. 4C).

**Fig. 5:**
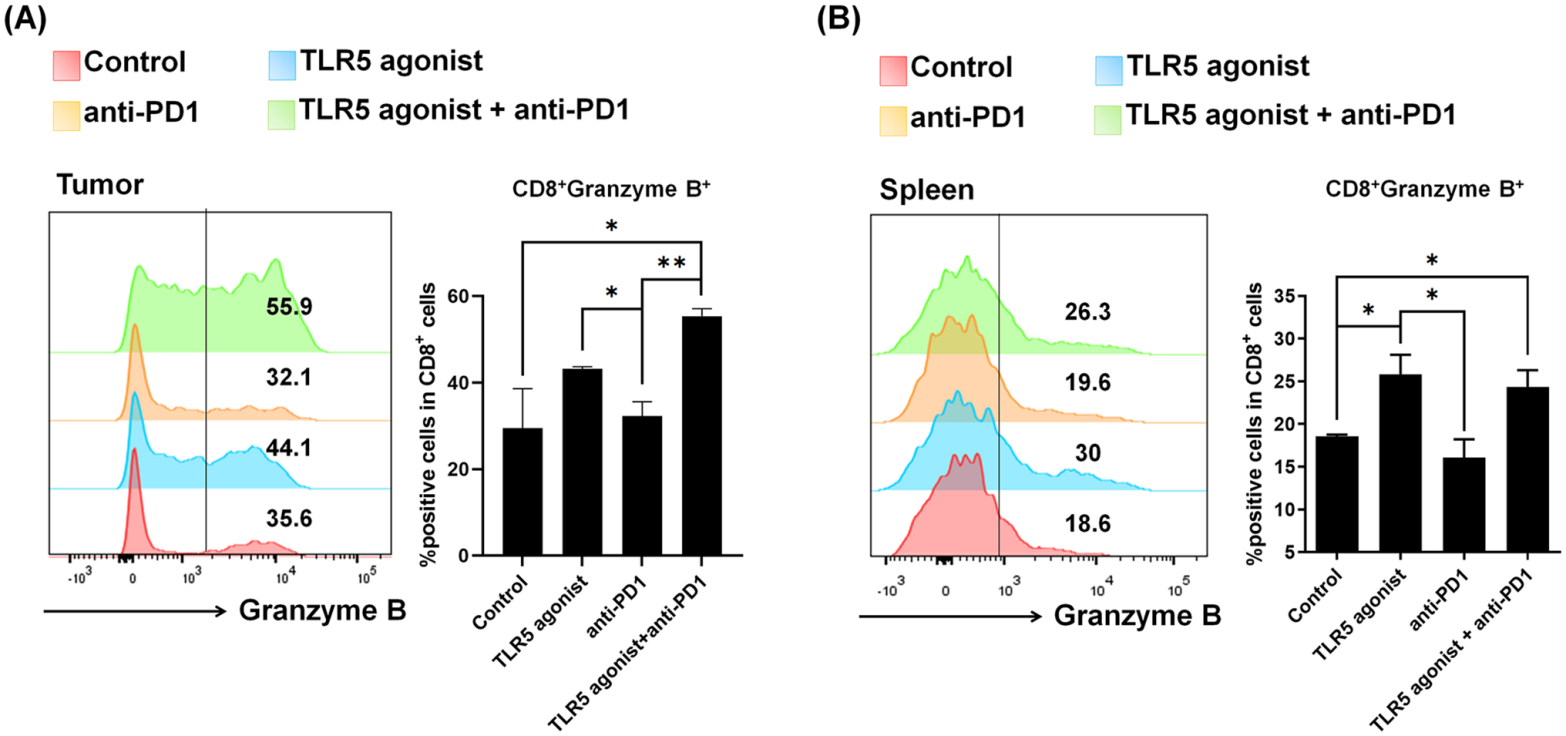
TLR5 agonist or combination treatment enhances activated CD8^+^ populations in the tumor and spleen. (A, B) Flow cytometry analyses of T cells (CD8^+^ granzyme B^+^) in **A** tumors and **B** spleens of tumor-bearing mice. Tumor tissues were sampled on day 11 after tumor implantation in the MC-38 tumor model. Spleen tissues were sampled on day 23 after tumor implantation in the MC-38 tumor model. Bars represent means ± SEM. *p < 0.05. The results are representative of three independent experiments (*n* = 3/group)

Following confirmation of CD8^+^ T cell activation by TLR5 agonist treatment, we explored the changes in granzyme B expression in CD8^+^ T and NK cells under each treatment. Flow cytometry of isolated tumor-infiltrating immune cells and splenocytes showed a notable increase in granzyme B within CD8^+^ T cells in both the TLR5 agonist monotherapy and combination therapy groups (Fig. 5A, B). For NK cells in the tumor, an increase in granzyme B was observed in the combination therapy group, whereas NK cells in the spleen showed no significant changes (Supplementary Fig. 6).

### ***Depletion of CD8***^+^***cells abrogated the anti-tumor effects of combination therapy on tumors***

**Fig. 6:**
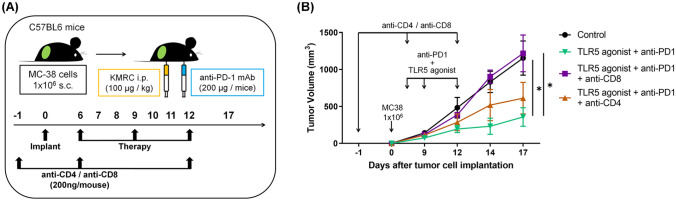
Depletion of CD8^+^ cells abrogated the anti-tumor effects of combination therapy on tumors. **A** MC-38 cells (1 × 106) were implanted subcutaneously (s.c.) in the flanks of C57BL/6 mice (*n* = 7/group). On day 6, mice were given the TLR5 agonist (100 µg/kg) and anti-PD-1 (200 µg/mouse), administered once every 3 days (three times in total). Anti-CD4 mAb or Anti-CD8 mAb was injected on days –1, 6, and 12. **B** Changes in tumor volume across groups. Tumor size was measured every 2–4 days starting on day 9. Bars indicate means ± SEM. *p < 0.05, **p < 0.01, ***p < 0.001

To confirm that tumor-specific CD8^+^ T cells induced by combination therapy contributed to suppressing the growth of distant metastatic tumors, CD8^+^ cells were depleted by anti-CD8 mAb. Depletion of CD8^+^ cells reversed the suppressive effects of the combination therapy (Fig. 6). Our results demonstrate that CD8^+^ T cells induced by combination therapy are critical for suppressing tumor growth. Additionally, although depletion of CD4^+^ cells weakened the inhibitory effect of combination therapy, the difference was not significant, suggesting a potential auxiliary role for CD4^+^ T cells in the anti-tumor immune response.

### TLR5 agonist or combination treatment enhances PD-L1 expression in TAMs

**Fig. 7:**
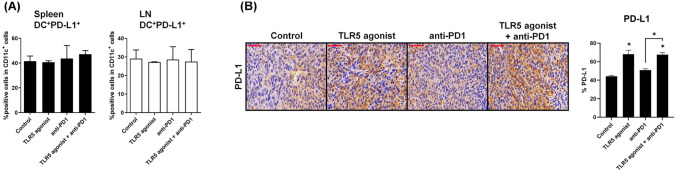
TLR5 agonist or combination treatment increases PD-L1 expression in TAMs. **A** Flow cytometry analyses of PD-L1 on DCs (CD11c^+^) in the spleen and LN of tumor-bearing mice. **B** Representative IHC staining of PD-L1 in tumors. Scale bars, 50 µm. Quantification of (mean ± SD) of PDL-1 is shown. Tumor and spleen tissues were sampled on day 23 after tumor implantation in the MC-38 tumor model. *p < 0.05. Results are representative of three independent experiments (*n* = 3/group)

Recent studies have suggested that the anti-tumor immune response elicited by immune checkpoint inhibitors might correlate with PD-L1 expression levels in tumor cells [28]. Thus, on day 26 after tumor implantation, we isolated spleens, LNs, and tumors to scrutinize the changes in PD-L1 expression in response to TLR5 agonists. Initial analysis of PD-L1 expression on levels in the spleen and LN revealed no marked differences among the treatment groups (Fig. 7A). However, IHC analysis of tumors showed increased PD-L1 expression in both the TLR5 agonist monotherapy and combination therapy groups (Fig. 7B). These findings indicate that TLR5 agonists could potentially modulate the anti-PD-1-triggered anti-tumor response by enhancing tumor PD-L1 expression.

## Discussion

We hypothesized that activation of innate immune cells by TLR5 agonists might enhance the effectiveness of immune checkpoint inhibitors by stimulating CD8^+^ T cell activation and promoting tumor infiltration. Using the MC-38 colorectal cancer mouse model, we examined the outcome of anti-tumor treatments using either TLR5 agonist or anti-PD-1 monotherapy, and both agents in combination. Our findings revealed that the combination therapy not only synergistically suppressed tumor growth but also elevated tumor necrosis. This supports the notion that TLR5 agonists augment the anti-tumor efficacy of anti-PD-1. To deepen our understanding of the role of TLR5 activation in tumor suppression, we examined the cytotoxic impact of spleen cells on MC-38 tumor cells following treatment with TLR5 agonist and anti-PD-1 (Supplementary Fig. 7). The absence of an effect of TLR5 agonist treatment in TLR5 knockout mice underscores the specificity of direct TLR5 activation by TLR5 agonists in tumor suppression. In light of the need for therapies targeting tumor patients resistant to immune checkpoint inhibitors [29, 30], we extended our investigation to include an anti-PD-1-unresponsive tumor model. We assessed the efficacy of TLR5 agonist and anti-PD-1 combination therapy in inhibiting tumor growth using the B16F10 tumor model. While anti-PD-1 monotherapy demonstrated no significant effect, TLR5 agonist monotherapy effectively inhibited tumor growth. Intriguingly, combination therapy involving the TLR5 agonist and anti-PD-1 exhibited a synergistic enhancement in the inhibitory effect on tumor growth. These findings suggest a potential strategy to augment the efficacy of immune checkpoint inhibitors, particularly in cases where their effectiveness is limited.

Both the TLR5 agonist monotherapy and the combination therapy promote a shift from M2-like macrophages to M1-like macrophages. M1 macrophages, which amplify inflammation and invigorate the immune system, obstruct tumor growth via substantial release of substantial pro-inflammatory cytokines and nitric oxide [31–33]. M1 macrophages have been shown to activate T cells through the upregulation of B7 molecules and antigen presentation via MHC class II molecules [34]. Recent research indicates that converting M2 macrophages back into M1 macrophages is essential to enhance the anti-tumor potency of immune checkpoint inhibitors [35]. Moreover, a higher M1/M2 ratio is consistently associated with a more favorable prognosis in tumor patients [36–39]. Based on this, we advocate exploration of TLR5 agonists as possible adjuvants in anti-tumor therapies, given their ability to raise the M1 polarization of macrophages.

Our analysis confirmed that TLR5 agonist monotherapy could attenuate the proliferation of MC-38 colorectal cancer cells, suggesting that treatment with a TLR5 agonist alone triggers an anti-tumor immune response against colorectal cancer. In xenograft and syngeneic mouse models of this cancer type, TLR5 signaling activation via TLR5 agonists can initiate innate immunity and elicit potent anti-tumor responses that regulate tumor growth [40]. TLR expression in colorectal cancer is intrinsically linked with its progression. An absence of TLR5 in tumor cells, as observed in mouse xenografts of human colorectal cancers, promotes tumor growth and reduces tumor necrosis [40, 41]. A recent study also highlighted a correlation between elevated TLR5 expression in tumor tissues and a more favorable prognosis for colorectal cancer patients [42]. Concerning the prevalent single-nucleotide polymorphisms (SNPs) in TLR5, research has been conducted to understand the link between TLR5 genotype and the survival rate of colorectal cancer patients [43]. These findings underscore the pivotal role TLR5 could play in advancing colorectal cancer diagnostics and treatments. Moreover, we confirmed the therapeutic potential of the TLR5 agonist and anti-PD-1 therapy in CT-26 colorectal cancer cells. We examined the cytotoxicity effects of splenocytes against the target CT-26 tumor cells after treatment of TLR5 agonist, anti-PD-1 antibody or both (Supplementary Fig. 8). We show that the combination treatment group significantly enhanced the anti-tumor effects compared to the single treatment groups. In conclusion, our data indicates that TLR5 agonists increase the M1-like polarization of macrophages and co-stimulatory molecule expression in macrophages. This leads to the activation of CD8 T cells and enhanced tumor invasion via TLR5 agonists. In essence, TLR5 acts in synergy with anti-PD-1 to suppress tumor growth. Our results highlight the potential of TLR5 to enhance the performance of immune checkpoint inhibitors.

### Supplementary Information

Below is the link to the electronic supplementary material.

Supplementary file 1 (DOCX 64545 kb)

## Data Availability

The datasets generated during and/or analyzed during the current study are available from the corresponding author on reasonable request.
