# Supplementary material for: TLR5 agonist in combination with anti-PD-1 treatment enhances anti-tumor effect through M1/M2 macrophage polarization shift and CD8+ T cell priming
Source: Cancer Immunol Immunother. 2024 Apr 17;73(6):102. doi: 10.1007/s00262-024-03679-5 (PMC11024077; doi:10.1007/s00262-024-03679-5)
Supplement: Supplementary file 1 — (DOCX 64545 kb) [file 262_2024_3679_MOESM1_ESM.docx]

**Supplementary figures**


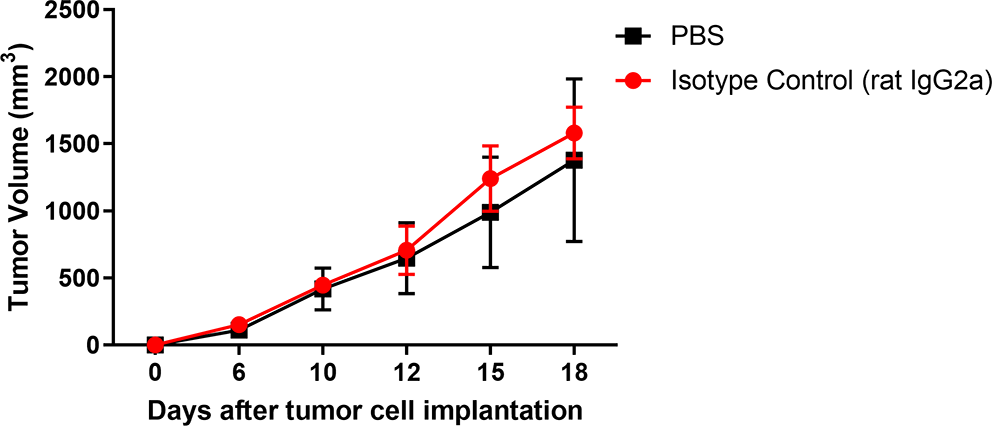


Supplementary Fig. 1 Influence of non-specific background signal from anti-PD1 antibody. MC-38 cells (1 × 10^6^) were subcutaneously (s.c.) implanted in the flanks of C57BL/6 mice(n=8/group). On day 6, mice received PBS or Rat IgG2a or κ isotype control mAb(200ug/mice), administered once every 3 days (three times in total). Changes in tumor volume across groups. Tumor size was measured every 2–4 days starting on day 6


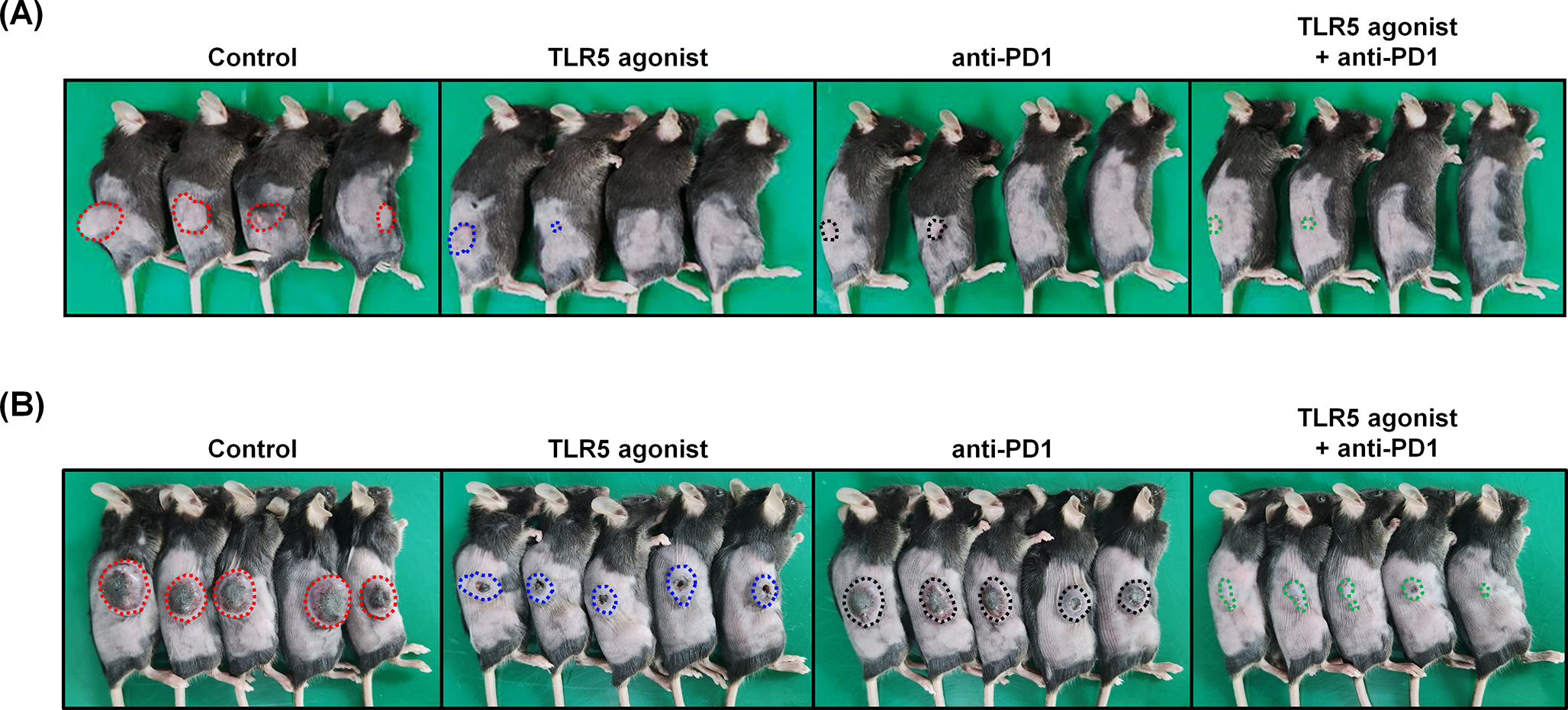


Supplementary Fig. 2 Combination therapy with TLR5 agonist and systemic anti–PD-1 antibody synergistically inhibits tumor growth. MC-38 or B16F10 cells (1 × 10^6^) were subcutaneously (s.c.) implanted in the flanks of C57BL/6 mice (n=8/group). On day 6, mice received either TLR5 agonist (100 µg/kg), anti-PD-1 (200 µg/mice), or a combination of both, administered once every 3 days (three times in total). (A) Photographs of representative mice 23 days after tumor implantation in the MC-38 tumor model. (B) Photographs of representative mice 17 days after tumor implantation in the B16F10 tumor model


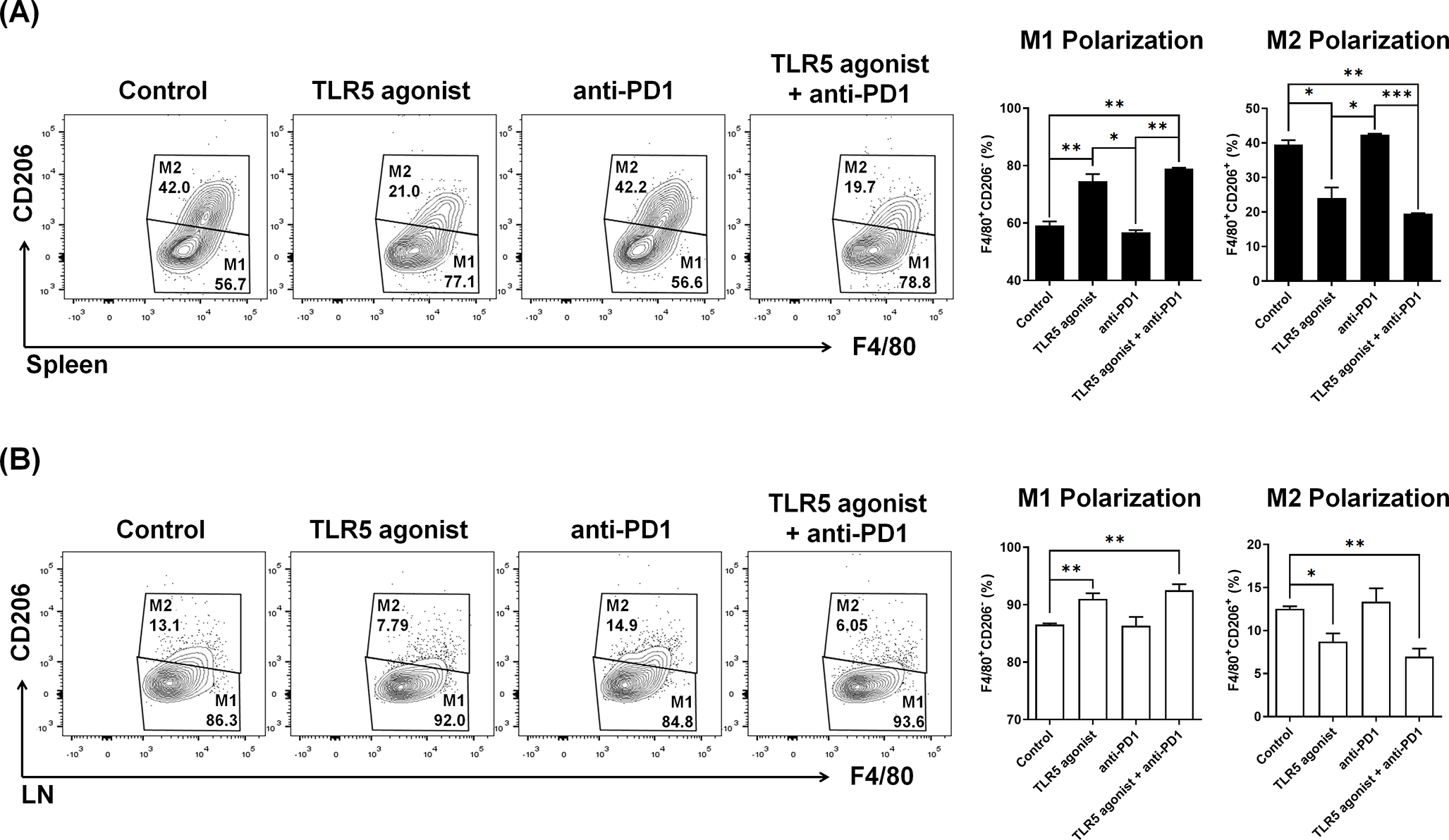


Supplementary Fig. 3 TLR5 agonist or combination treatment promote a shift from M2-like macrophages to M1-like macrophages in the spleen and lymph node. (A, B) Flow cytometry analyses of M1-like (F4/80^+^/CD206^-^) and M2-like (F4/80^+^/CD206^+^) macrophages in (A) spleen and (B) LN from tumor-bearing mice. Specimens were collected on day 23 after tumor implantation in the MC-38 tumor model. Bars represent means ± SEM. *p < 0.05, **p < 0.01, ***p < 0.001. Results are representative of three independent experiments (n=3/group)


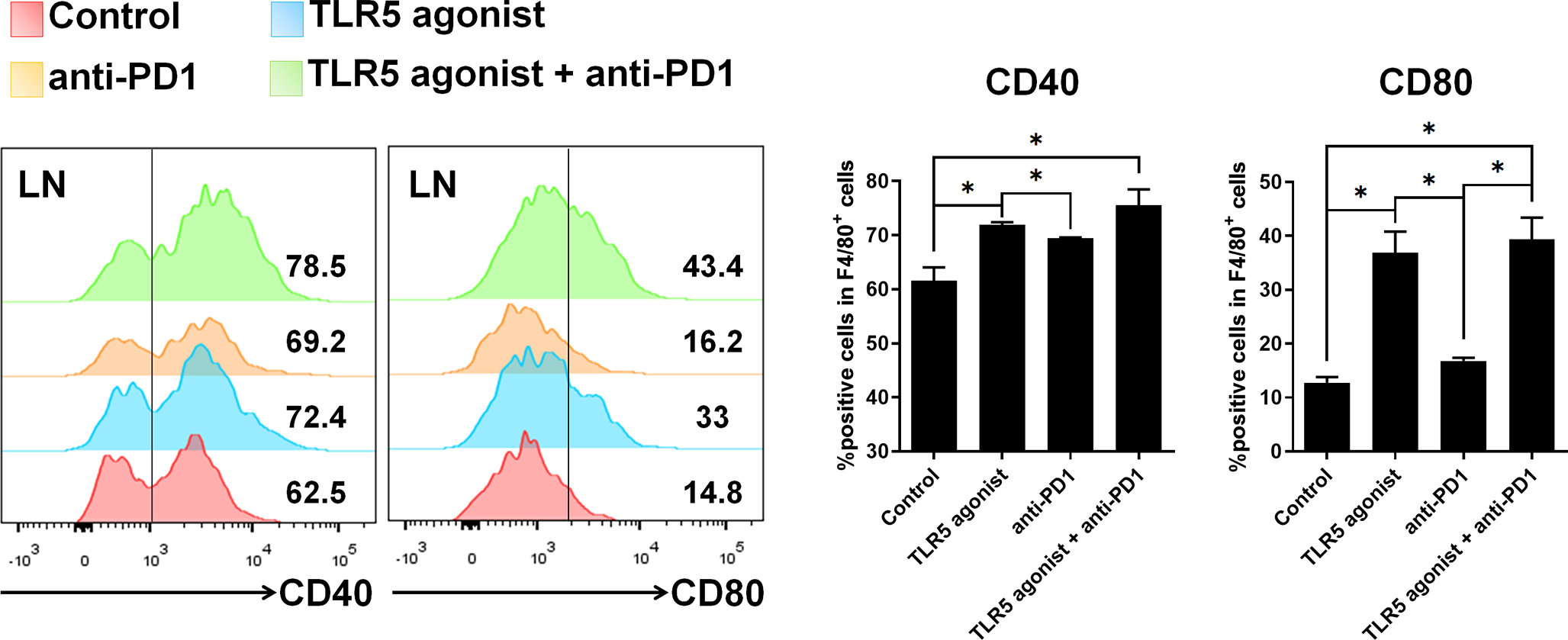


Supplementary Fig. 4 TLR5 agonist or combination treatment elevates costimulatory molecule expression in macrophages. Flow cytometry analyses of costimulatory molecules (CD40 and CD80) on macrophages from the LN tissues of tumor-bearing mice. LN tissues were sampled on day 23 after tumor implantation in the MC-38 tumor model. Bars represent means ± SEM. *p < 0.05, **p < 0.01, ***p < 0.001. The results are representative of three independent experiments (n = 3/group)


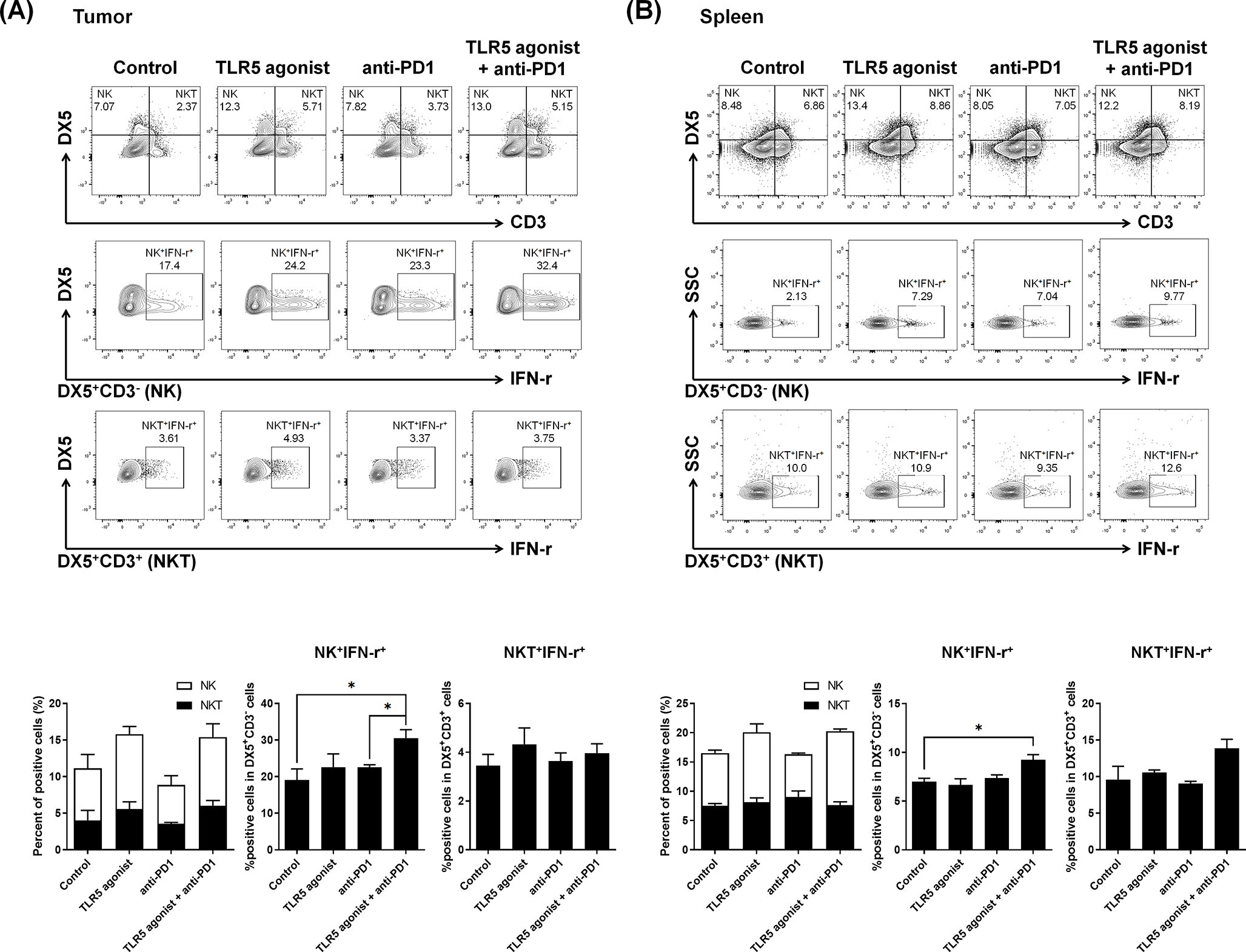


Supplementary Fig. 5 TLR5 agonist or combination treatment increases the activated NK cells in the tumor and spleen. (A, B) Flow cytometry analyses of NK cells (DX5^+^ IFN-γ^+^) and NKT cells (DX5^+^CD3^+^IFN-γ^+^) in (A) tumors and (B) spleens of tumor-bearing mice. Tumor tissues were sampled on day 11 after tumor implantation in the MC-38 tumor model. Spleen tissues were sampled on day 23 after tumor implantation in the MC-38 tumor model. Bars indicate means ± SEM. *p < 0.05, **p < 0.01. The results are representative of three independent experiments (n = 3/group)


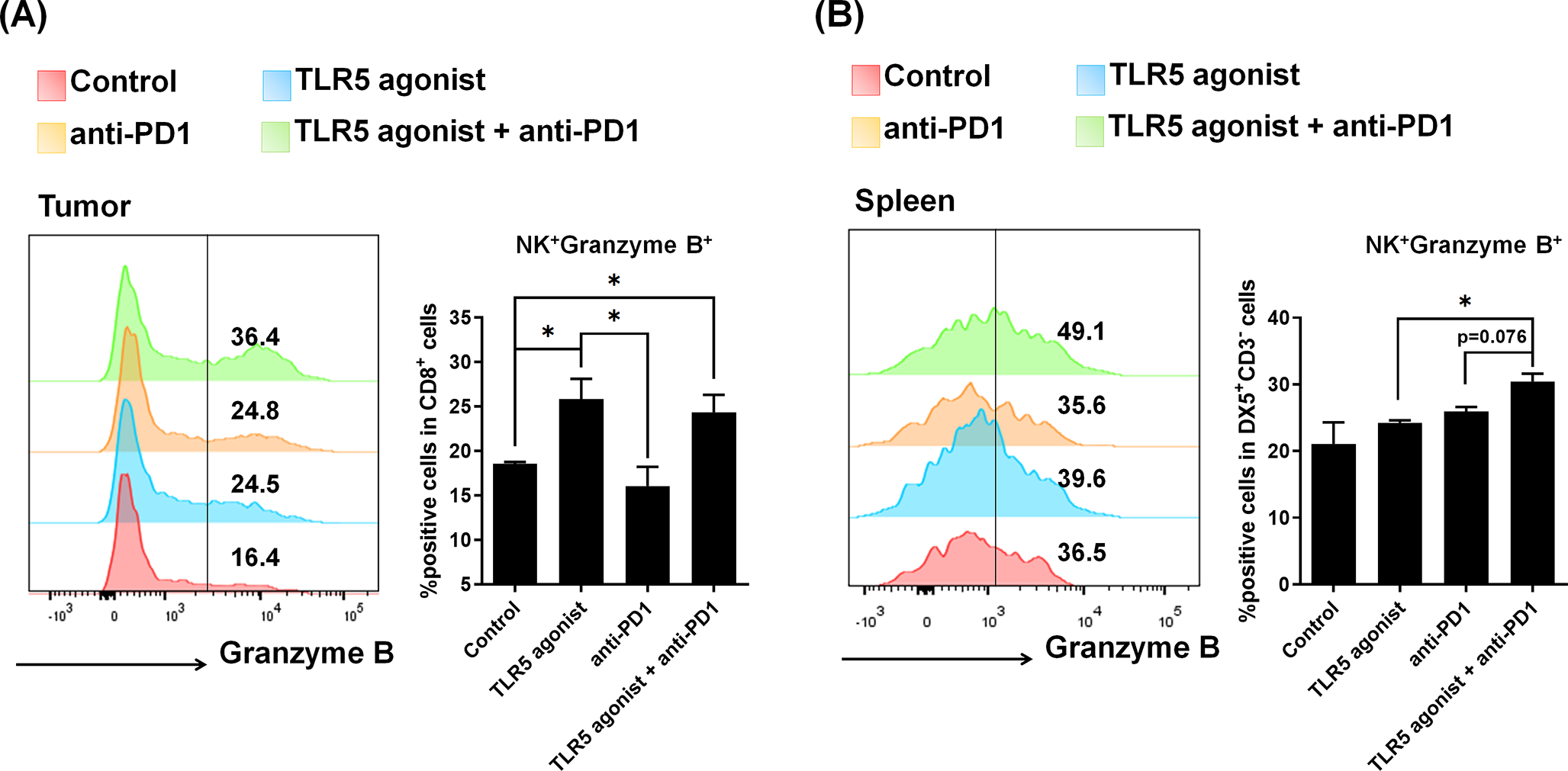


Supplementary Fig. 6 TLR5 agonist or combination treatment enhances activated NK cells in the tumor and spleen. (A, B) Flow cytometry analyses of NK cells (DX5^+^ granzyme B^+^) in (A) tumors and (B) spleens of tumor-bearing mice. Tumor tissues were sampled on day 11 after tumor implantation in the MC-38 tumor model. Spleen tissues were sampled on day 23 after tumor implantation in the MC-38 tumor model. Bars represent means ± SEM. *p < 0.05. The results are representative of three independent experiments (n = 3/group)


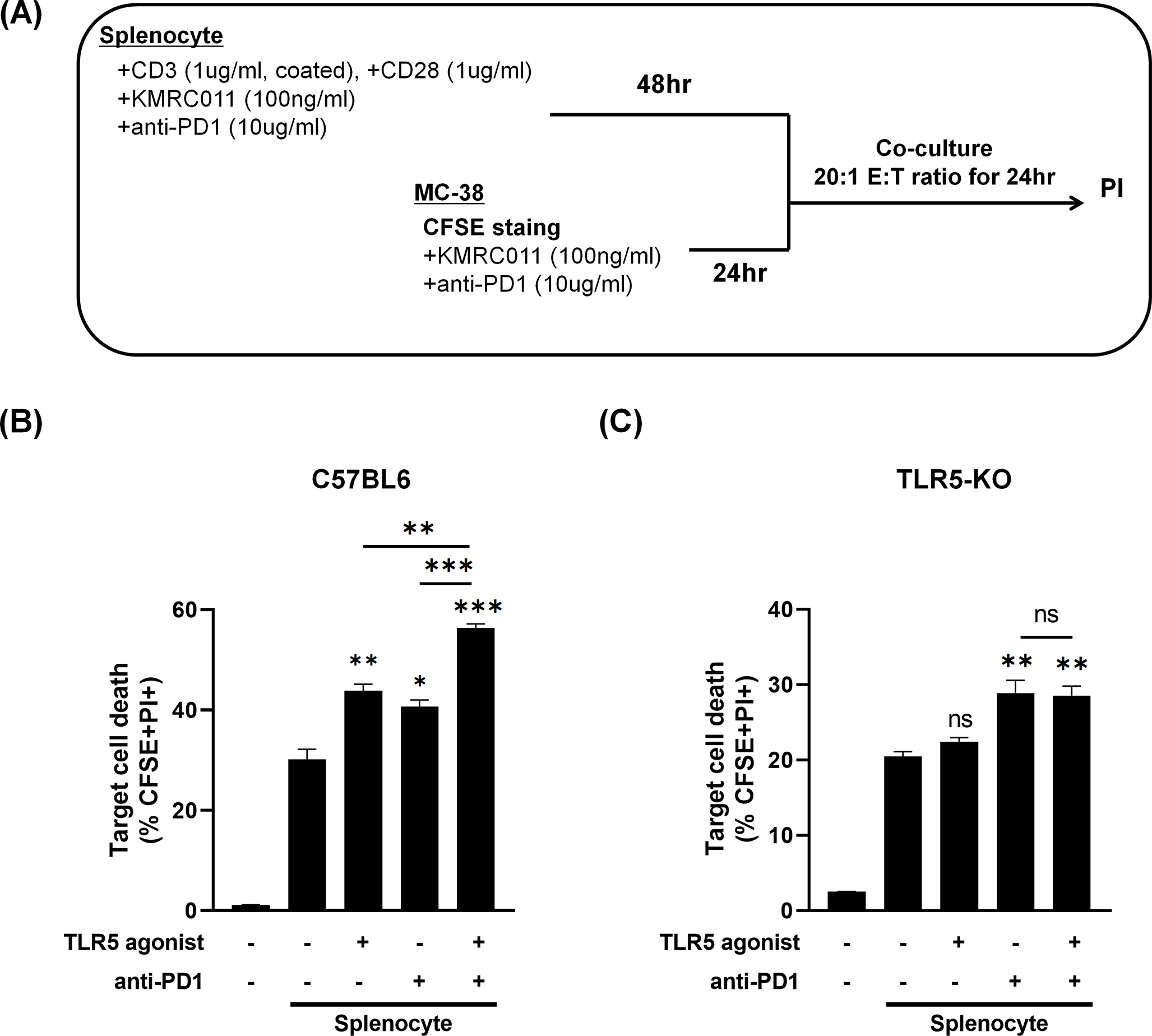


Supplementary Fig. 7 TLR5 agonists suppress tumors by mediating TLR5 activation. (A) C57BL/6 and TLR-KO mouse splenocytes were stimulated with anti-CD28 antibody(1µg/mL) and anti-CD3 antibody (1µg/mL) for 48h in the presence of TLR5 agonist (100ng/ml) and/or anti-PD1 abs (10ug/ml) (or vehicle treatments). MC-38 tumor cells stained with CellTrace CFSE (5μM) (Thermo Fisher) and treated with TLR5 agonist (100ng/ml) and/or anti-PD1 abs (10ug/ml) (or vehicle treatments) for 24hr. Pre-treated splenocytes and MC-38 tumor cells were co-cultured at a 1:20 (target:effector) ratio for 24 hours. (B, C) The killing function of splenocytes from (B) C57BL/6 and (C) TLR-KO mice against MC-38 tumor cells as target cells (mean % MC-38 cell death ± SEM for n = 3) was quantified through flow cytometry. Bars represent means ± SEM. The summary of P values for each group compared to the splenocyte-only group, which did not receive treatment with TLR agonist and anti-PD1, is presented above the bar. *p < 0.05, **p < 0.01, ***p < 0.001


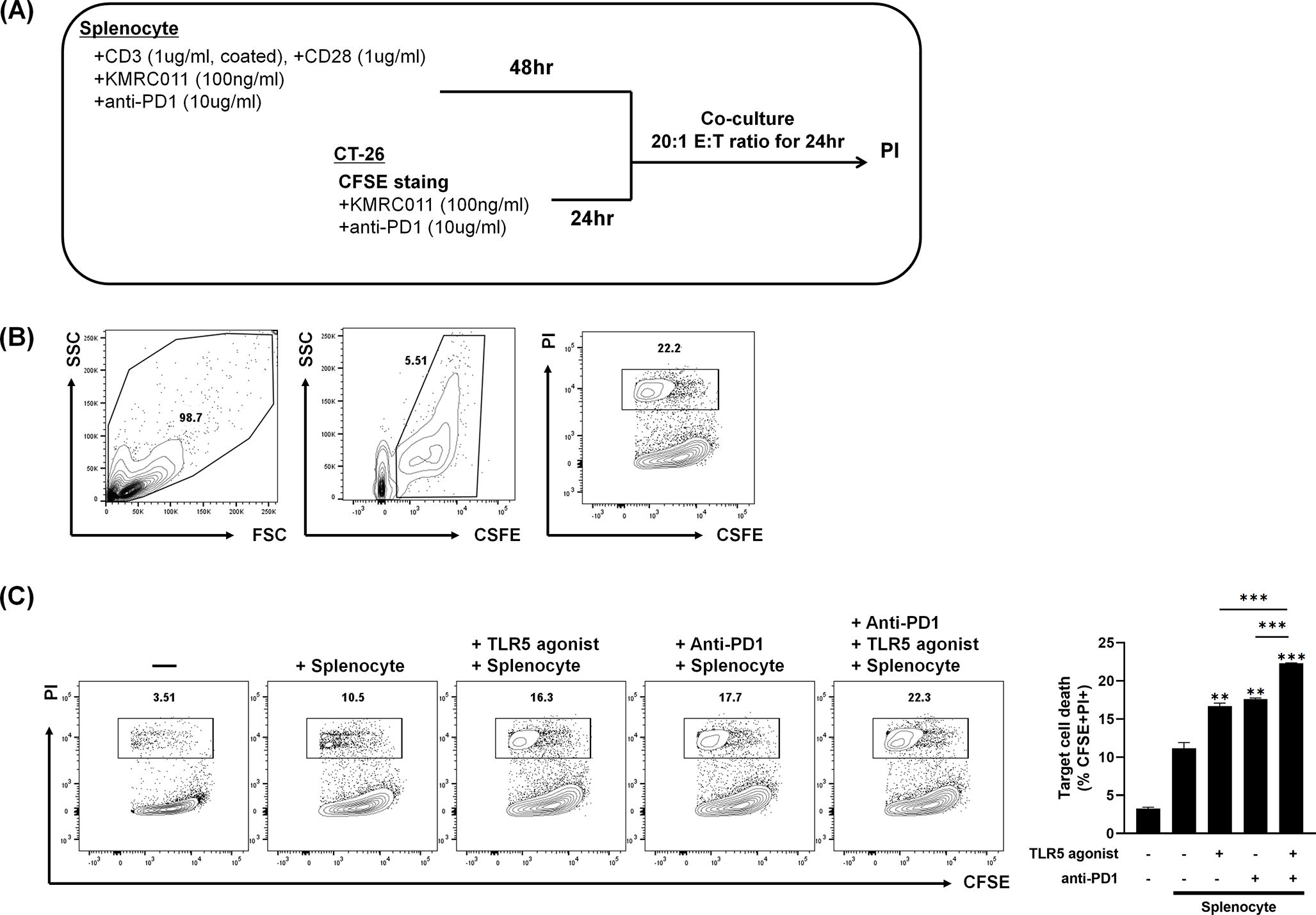


Supplementary Fig. 8 Combination TLR5 agonist with anti-PD-1 abs effectively reactivates anti-tumor splenocytes killing function. (A) BALB/c mouse Splenocytes were stimulated with anti-CD28 antibody(1µg/mL) and anti-CD3 antibody (1µg/mL) for 48h in the presence of TLR5 agonist (100ng/ml) and/or anti-PD1 abs (10ug/ml) (or vehicle treatments). CT26 tumor cells stained with CellTrace CFSE (5μM) (Thermo Fisher) and treated with TLR5 agonist (100ng/ml) and/or anti-PD1 abs (10ug/ml) (or vehicle treatments) for 24hr. Pre-treated Splenocytes and CT26 tumor cells were co-cultured at a 1:20 (target:effector) ratio for 24 hours. The splenocytes killing function against CT26 tumor cells as target cells (mean % CT26 cell death ± SEM for n = 3) was quantified through flow cytometry. (B) Representative images of gating schematic. CT26 cell death was identified through CFSE and PI staining. (C) The representative degree of CT26 cell death in each group analyzed by flow cytometry is indicated. Bars represent means ± SEM. The summary of P values for each group compared to the splenocyte-only group, which did not receive treatment with TLR agonist and anti-PD1, is presented above the bar. *p < 0.05, **p < 0.01, ***p < 0.001
